# Food as Treatment of Inflammatory Bowel Diseases

**DOI:** 10.1128/iai.00583-21

**Published:** 2022-05-11

**Authors:** Ana Maldonado-Contreras

**Affiliations:** a University of Massachusetts Chan Medical School, Department of Microbiology and Physiological Systems, Program of Microbiome Dynamics, Worcester, Massachusetts, USA; University of California, Santa Cruz

**Keywords:** diet, epithelial barrier, gut inflammation, inflammatory bowel disease, microbiome

## Abstract

Inflammatory bowel diseases (IBD), namely, Crohn’s disease (CD) and ulcerative colitis (UC), are lifelong and incurable chronic inflammatory diseases affecting 6.8 million people worldwide. By 2030, the prevalence of IBD is estimated to reach 1% of the population in Western countries, and thus there is an urgent need to develop effective therapies to reduce the burden of this disease. Microbiome dysbiosis is at the heart of the IBD pathophysiology, and current research and development efforts for IBD treatments have been focused on gut microbiome regulation. Diet can shape the intestinal microbiome. Diet is also preferred over medication, is safe, and has been proven to be an effective strategy for the management of IBD. Therefore, although often overlooked, dietary interventions targeting the microbiome represent ideal treatments for IBD. Here, I summarize the latest research on diet as a treatment for IBD from infancy to adulthood, compile evidence of the mechanisms of action behind diet as treatment, and, lastly, provide insights into future research focusing on culturally tailored diets for ethnic minority groups with increased incidence of IBD yet underrepresented in nutrition research.

## INTRODUCTION

The incidence of inflammatory bowel disease (IBD) is on the rise (1). In the United States alone, around 2.5 million people suffer from IBD, resulting in up to $31.6 billion in direct and indirect costs annually (2–4). Despite the multifactorial causality of IBD, dysbiosis, epithelial barrier dysfunction, and immune disturbances have been suggested as the cornerstones of disease onset and severity (5–9). Most of the current treatments rely on pharmacological interventions that either dampen inflammation (i.e., corticosteroids, immunosuppressants) or decrease the chance of bacterial breach from the lumen to the underlying mucosa due to a dysfunctional epithelial barrier (i.e., antibiotics). These conventional treatments have remission rates lower than 50% and usually fail to prevent recurrent flare-ups over time. More than one-third of patients with IBD fail induction therapy, and up to 60% of primary responders lose response over time (10–12). With a rising prevalence of IBD worldwide (13), there is an urgent need to develop effective and sustainable therapies that can be used long-term.

Interestingly, a microbiome “imbalance” or dysbiosis is linked to barrier dysfunction (14–17) and inflammation (18–21), which together are responsible for the IBD pathophysiology (8, 22). Over the last decade or so, there has been an increasing enthusiasm about clinical applications of diet as microbiome-targeted therapy as an adjunct treatment of IBD. Diet is a modifiable, noninvasive, inexpensive behavior that is crucial in shaping the intestinal microbiome (23–28). Dietary patterns have been associated with increased IBD risk or with the characteristic dysbiosis found in IBD patients in several large cohorts (29–33). Thus, diet can serve as a microbiome-targeted adjunct therapy to assist in the management of IBD. To date, intervention studies have shown that diet, in conjunction with medication, is effective in inducing IBD remission and can be tolerated without adverse effects (34–45). This review aims to summarize results from studies assessing (i) diet as therapy for IBD, (ii) its effect on the microbiome of patients with IBD, and (iii) the microbiota-dependent mechanisms by which food affects IBD outcomes.

## DIETARY TREATMENT FOR IBD ACROSS THE LIFE SPAN

### Early life.

Since the pioneering analysis of the Swedish twin registry, mounting evidence demonstrates a robust genetic basis of IBD (46). However, genetics explain only a minority of the variance of disease risk, suggesting that a combination of genetics and environmental factors will be more likely to explain disease pathogenesis. For instance, infants born to mothers with IBD are at a substantially increased risk of developing the disease compared to infants born to fathers with IBD, particularly Crohn’s disease (CD) (47). Akolkar et al. found that out of 135 families where both a parent and a child had IBD, the parent-to-child transmission was linked to the mother in 69% of cases and only 31% of the cases were linked to the father having IBD (48). A second study found a similar trend, with mother-to-child transmissions accounting for 63% of Crohn’s disease cases (49). Finally, studies of children who subsequently developed IBD showed an increased risk (7 to 8 times higher) of Crohn’s disease if the mothers also suffered from the disease (47, 50).

Hence, besides genetics, a possible explanation of the higher proportion of mother-to-child than father-to-child transmissions is that environmental factors during pregnancy might affect the risk of developing IBD. Accumulating evidence suggests that the “inherited” neonate microbiome from the mother can exert marked effects on the immune and metabolic programming of the offspring, with long-term health-related consequences including the predisposition to IBD (51–54). Environmental factors during pregnancy, including mode of delivery, perinatal diet, and perinatal antibiotic use, drive the composition of the “inherited” neonate microbiome (55, 56). For instance, infants born by cesarean exhibit a neonate microbiome with low diversity and late colonization of *Bacteroidetes* that is associated with reduced Th1 cell responses in the first 2 years of life (57). Activation of Th1 triggers the cellular immune response, inhibits macrophage activation, and stimulates B cells to produce IgM and IgG1 antibodies. In addition, experiments in mice have demonstrated that the neonate microbiome can prevent the accumulation of invariant natural killer T cells (iNKT), which results in decreased disease severity in models of IBD (58).

Infants born to mothers with IBD exhibit a higher abundance of *Gammaproteobacteria* species and depletion of *Bifidobacterium* species than those born from mothers without IBD (59). Furthermore, maternal IBD status is a significant predictor of the overall β-diversity of the neonate microbiome and of the abundance of bifidobacteria and *Gammaproteobacteria* over time. Infants born to mothers with IBD had higher levels of fecal calprotectin (FC), an inflammatory marker that robustly correlates with barrier damage and massive neutrophil infiltration in IBD patients (60–65). The levels of fecal calprotectin in those babies are higher as early as 2 months and up to 36 months of age than the levels in babies born to healthy mothers (59, 66). Specific bacteria were correlated with fecal calprotectin levels in the infants; namely, *Bifidobacterium* (depleted in infants born from mothers with IBD), *Faecalibacterium*, and *Alistipes* showed negative correlations, and Streptococcus was positively correlated with fecal calprotectin levels within 3 months of birth (66). Experiments with germfree mice inoculated with stools of mothers with IBD showed that mice developed an altered immune maturation. Particularly, mice inoculated with stools of mothers with IBD showed significantly lower levels of switched memory B cells (CD19^+^ CD27^+^ IgM^−^ IgD^−^) and regulatory T cells (CD3^+^ CD4^+^ FoxP3^+^) than did germfree mice inoculated with stools from pregnant controls (59). The long-term consequences of elevated fecal calprotectin levels or/and an altered immune maturation at an early age are uncertain and hard to evaluate. Nonetheless, restoration of the microbiome in mothers with IBD or their infants can retune the microbiome-dependent immune and metabolic programming of the offspring.

Diet can rapidly and predictably change the microbiome (23). It has been demonstrated that perinatal diet is linked to the “inherited” neonate microbiome (67–69). Lundgren et al. analyzed the stool microbiome of 145 infants enrolled in the New Hampshire Birth Cohort Study and compared it to the dietary information obtained during pregnancy from their mothers using a food frequency questionnaire (67). They found that the microbiome of infants born vaginally grouped into three clusters, each cluster dominated by either (i) *Bifidobacterium*, (ii) Streptococcus and *Clostridium*, or (iii) *Bacteroides*. The odds of belonging to the Streptococcus*-* and *Clostridium-*dominated cluster was 2.73 times greater for each additional maternal serving of fruit per day. Other foods consumed during pregnancy are associated with specific members of the microbiome in the offspring. For example, high dairy intake during pregnancy was positively associated with Clostridium neonatale, Clostridium butyricum, and Staphylococcus and negatively related to *Lachnospiraceae* species (67). Another study of mothers in the Spanish-Mediterranean area (*n* = 116 mothers) showed that the maternal microbiome of this cohort could be grouped into two clusters: *Prevotella*-dominated cluster I and *Ruminococcus*-dominated cluster II (69). Mothers in the *Ruminococcus*-dominated cluster II reported higher intakes of total dietary fiber, monounsaturated omega-3 fatty acids, and polyphenols, while mothers in the *Prevotella*-dominated cluster I reported higher consumption of carbohydrate and saturated fatty acids (SFA). The authors found that maternal clustering of the microbiome correlated with the neonatal microbiome composition (*n* = 86 dyads). Infants born from mothers in the *Prevotella*-dominated cluster I exhibited a higher abundance of *Firmicutes* spp. than those born from mothers in the *Ruminococcus*-dominated cluster II. Besides the neonatal microbiome, clustering of the maternal microbiome was also linked to the risk of being overweight. Validated anthropometrical measurements such as weight-for-length (WFL) and body mass index (BMI) Z-scores were collected from infants up to 18 months of age to assess the risk of being overweight. Infants belonging to mothers in the *Prevotella*-dominated cluster I—with higher consumption of carbohydrate and SAF—and born by cesarean section exhibited significantly higher BMI and WFL Z-scores at 18 months than infants from vaginal births from mothers classified in the *Ruminococcus*-dominated cluster II—with higher intakes of total dietary fiber, monounsaturated omega-3 fatty acids, and polyphenols (69). Later, Selma-Royo et al. reported a subsequent study including 73 dyads from the same Spanish cohort (68). Here, they showed that intake of SFA and monounsaturated fatty acids (MUFA) during pregnancy significantly affects the overall structure of the offspring microbiome. Particularly, infants of mothers with high SFA and MUFA intakes showed enrichment in *Firmicutes* (i.e., *Coprococcus*, *Blautia*, *Roseburia*, *Ruminococcaceae*, and *Lachnospiraceae*) and depletion in *Proteobacteria* (i.e., Klebsiella, Escherichia). Conversely, intakes of vegetable-derived proteins and fiber during pregnancy negatively correlate with enrichment of the *Firmicutes* species mentioned above in the offspring (68).

To the best of my knowledge, only one study in Norway has delved into the dietary patterns of pregnant women with IBD (70). The authors of the study enrolled 183 mothers with CD, 240 mothers with ulcerative colitis (UC), and 83,565 mothers without IBDs. They found that mothers with IBD had low adherence to a “traditional dietary pattern,” characterized by consumption of lean fish, fish products, potatoes, rice porridge, cooked vegetables, and gravy. High adherence to the traditional dietary pattern was associated with improved pregnancy outcomes, namely, a lower risk for “small for gestational age” outcome (70).

My laboratory, in collaboration with researchers at Icahn School of Medicine at Mount Sinai, is currently testing a dietary intervention in mothers with IBD to revert dysbiosis during pregnancy and, in consequence, the “inherited” neonate microbiome and early inflammation seen in infants born to mothers with IBD (45).

### Pediatrics.

Exclusive enteral nutrition (EEN) is the primary therapy used to induce remission in pediatric IBD patients, particularly CD (71). This therapy consists of replacing foods with commercial formulas (elemental or polymeric) to provide total calories, complete macronutrients, and micronutrients to pediatric patients with active disease. EEN induces remission in 80 to 85% of pediatric patients, similar to those treated with corticosteroids (72). Compared to steroid treatment, several studies have demonstrated that EEN is superior in inducing mucosal healing in pediatric patients (73–77). However, a meta-analysis confirmed that corticosteroid therapy in adults with active disease may be more effective in inducing remission than EEN (78). Thus, EEN is used primarily in pediatric patients.

EEN drives microbiome changes. Specifically, EEN results in a reduction of bacterial diversity along with a decreased abundance of specific short-chain fatty acid (SCFA)-producing bacteria thought to be beneficial (i.e., *Faecalibacterium*, *Ruminoccocus*, *Blautia*, and *Subdoligranulum*) (79, 80). Moreover, EEN stabilizes the microbiota-dependent metabolism of bile acids (BAs) (80). BAs play a central role in modulating intestinal immune responses and possess antimicrobial activity that can inhibit bacterial overgrowth (81). The liver produces primary BAs from cholesterol, and the gut microbiome can modify these compounds into a myriad of secondary BAs that greatly increase their diversity and biological function (82–85). A recent study investigated the BA proportions in fecal samples obtained from 17 CD children before and after EEN treatment. Six of the 17 children did not sustain remission while on EEN treatment and experienced a relapse requiring escalation of medical therapy (e.g., oral corticosteroids, biologic therapy, or surgery). The six children experiencing relapses showed an accumulation of primary BAs in stool before EEN treatment, suggesting depletion of bacteria capable of generating secondary BAs. Those children exhibited a significantly higher abundance of multiple bacteria species unable to modify BAs (i.e., Bacteroides plebeius, Bacteroides ovatus, Bacteroides dorei, Bacteroides thetaiotaomicron, Ruminococcus gnavus, Escherichia coli, Clostridium bolteae, and *Veillonella* sp.). Conversely, children with EEN-sustained remission (*n* = 7) exhibited accumulation of microbiome-dependent secondary BAs before EEN and at any given time point (86). Samples from children with EEN-sustained remission showed enrichment of bacterial species with the genetic capacity to modify primary BAs into secondary BAs (i.e., Bacteroides vulgatus, Bacteroides uniformis, *Faecalibacterium*, *Subdoligranulum*, and *Alistipes* sp.) (86). This suggests that microbiota-dependent production of secondary BAs is necessary for EEN-dependent sustained remission.

Other diets tested as a therapy for pediatric patients include the specific carbohydrate diet (SCD) (36, 41), the modified SCD (mSCD) (36), Crohn’s disease treatment with eating (CD-TREAT) (42), and the Crohn’s disease exclusion diet (CDED) (34, 40, 44).

The SCD is one of the most studied diets in the IBD population. SCD focuses on removing grains (i.e., wheat, oats, barley, corn, quinoa, and rice) and milk products (except for hard cheeses and fully fermented yogurts) and replacing any added sugar with honey. Hence, SCD is centered on low complex carbohydrates that can serve as food sources for beneficial bacteria in the colon, thus reverting dysbiosis and reducing inflammation (41). The first study in children with CD (*n* = 7) showed improvement of inflammatory markers after several months of SCD treatment (41). A few retrospective studies have also confirmed that pediatric patients with CD or UC experienced a reduction of disease activity after SCD treatment (87, 88). Later, a prospective study of children with mild to moderate IBD showed that adding a 12-week treatment with SCD along with concurrent medication induced remission in 8 of the 12 patients included in the study (89). Despite the small sample size and high interpersonal variability, children in the study showed changes in the microbiome, with *Bacteroides* and *Parabacteroides* having the largest decrease in median abundance and *Eubacterium*, *Ruminoccocus*, and *Subdoligranulum* the largest increase (89). Of note, depletion of *Eubacterium*, *Ruminoccocus*, and *Subdoligranulum* has been associated with dysbiosis in IBD patients (90). A more recent randomized trial compared the SCD, the mSCD (integrating some initially excluded foods, such as potatoes, rice, quinoa, and oats), and whole foods (eliminating wheat, corn, sugar, milk, and food additives) (36). Here, researchers found that all the children (*n* = 10) completing 12 weeks on any one of the treatments achieved remission. As expected, children exhibited a microbiome shift that was primarily patient specific (36).

Of note, nutritional deficiencies are common in IBD individuals (i.e., low in calcium, iron, vitamins B6, B9, and B12, vitamin D, and others) (91–93). In children with IBD, these deficiencies can lead to growth failure and malnutrition, which are among the major complications of the disease (94). Both EEN and SCD are restrictive diets that are recommended for short-term consumption, as nutritional deficiencies may arise (95, 96).

On that note, investigators in the United Kingdom created a less-restrictive diet for pediatric patients, CD-TREAT (42). CD-TREAT is a diet based on solid food, aimed to recapitulate the nutrient composition and effects of EEN in the microbiome. An initial study on healthy volunteers (*n* = 25) demonstrated that CD-TREAT is better tolerated than EEN, with similar effects on the microbiome composition and metabolic function (42). Specifically, individuals on either CD-TREAT or EEN showed marked reductions of short-chain fatty acid (SCFA)-producing bacteria, along with lower concentrations of SCFAs in feces. The microbial genetic capacity for BA modification was not assessed in this study. CD-TREAT was then tested in an open-label trial on 5 children with CD who had mild to moderate active luminal disease. Four children completed the study. After 4 weeks on CD-TREAT, 3 patients demonstrated a clinical response (weighted pediatric Crohn’s disease activity index [wPCDAI] score change, >17.5) and 2 were in clinical remission (wPCDAI score, <12.5). At 8 weeks, all the patients showed a clinical response and 3 entered clinical remission (42).

Another whole-food diet, the CDED, was designed to exclude potentially proinflammatory food ingredients, such as gluten, dairy products, gluten-free baked goods and bread, animal fat, processed meats, products containing emulsifiers, canned goods, and all packaged/processed products (44). CDEDs have been tested coupled with partial enteral nutrition (CDED+PEN) but not alone. The first report showed that 33 of 47 children (70%) treated with CDED+PEN for 6 weeks achieved clinical remission (44). In a second study, researchers compared EEN to CDED+PEN and found that the latter is better tolerated by pediatric patients (73.6% versus 97.5%) (34). At week 6, 30 (75%) of 40 children treated with CDED+PEN were in corticosteroid-free remission versus 20 (59%) of 34 children given EEN. The authors assessed the microbiome compositions of 28 pediatric patients on the CDED+PEN and 32 on the EEN. They observed a significant reduction of *Actinobacteria* and *Proteobacteria* species and an increased abundance of *Clostridia* sp. after 6 weeks on either diet. At week 12, patients on the CDED+PEN maintained the dominance of *Clostridia* and the decrease of *Proteobacteria* with a minor rebound of *Actinobacteria*, while patients on the EEN reverted to the pretreatment microbiome composition (34). The latest multicenter randomized trial of CDED+PEN versus EEN in children with mild to moderate CD showed that either treatment resulted in 63% and 67% remission (pediatric Crohn’s disease activity index [PCDAI], <10) rates after 3 and 6 weeks of treatment, respectively (40). See Table 1 for a summary of the trials discussed above.

**TABLE 1:** Summary of dietary treatments for IBD tested in human trials

Diet(s) tested (reference)	Population tested	Study design	No. of subjects included	Duration of the intervention	Outcomes^*a*^
EEN (polymeric diet) vs corticosteroids (73)	Children with active, naive CD	Prospective, randomized, open-label trial	*n* = 37, 19 on the polymeric diet and 18 on corticosteroids	10 wk	Remission in 79% of patients on EEN group compared to 67% of patients on corticosteroid group. Mucosa healing was significantly higher in the EEN group, 74%, than in the corticosteriod group, 33%.
EEN (polymeric diet, semi-elemental diet, and elemental diet) vs corticosteroids (74)	Children with newly diagnosed active CD	Retrospective	*n* = 47, 37 on EEN (12 polymeric; 13 semi-elemental; 12 elemental diet) and 10 on corticosteroids	8 wk	Remission in 86.5% patients on EEN vs 90% treated with corticosteroids. Mucosa healing was higher in the EEN group, 64.8%, than in the corticosteroid group, 40%. Compared to group receiving corticosteroids, the duration of clinical remission was longer in the EEN groups, without differences among the three different formulas.
EEN (79)	Children with active CD	Prospective, nonblinded observational case study with both groups receiving the intervention	*n* = 44, 23 patients with CD and 21 controls	8 wk	Remission in 62% of patients with CD. Reduction of bacterial diversity. Reduction of *Bifidobacterium, Ruminococcus,* and *Faecalibacterium.*
EEN (80)	Children newly diagnosed with CD	Prospective, nonblinded observational case study with only CD patients receiving the intervention. Stool sample collected prior to, during, and after EEN.	*n* = 43	6 wk	During the intervention, there was a decrease in microbiota diversity and a reduction of amino acids. Also, an increase in microbial metabolism of bile acids. Prior to EEN, microbiota and metabolome are different between responders and nonresponders
SCD (41)	Children with CD (PCDAI > 10)	Retrospective	*n* = 7 patients with CD	5–30 mo	All symptoms resolved 3 mo after SCD. Serum albumin, C-reactive protein, hematocrit, and stool calprotectin either normalized or significantly improved during follow-up clinic visits.
SCD vs mSCD vs whole foods (36)	Children with CD, mild to moderate	Randomized trial	*n* = 14, 5 on the SCD, 5 on the mSCD, and 4 on the whole food diet. Only 10 completed the study.	12 wk	100% of patients on any of the diets achieved remission. At wk 12, 100% of participants who had elevated CRP at enrollment (*n* = 8) and completed the study had normal CRP, and 80% (*n* = 8 out of 10) of participants had a decrease in ESR.
CD-TREAT (42)	Children with CD, mild to moderate active luminal disease	Prospective, open-label trial	*n* = 5 (only 4 completed the study)	4 wk, with additional 4 wk (follow-up)	3 out of 4 children achieved clinical response, 2 achieved remission (4 wk). All patients achieved clinical response, 3 achieved remission (8 wk).
CDED+PEN or CDED alone (44)	Children and young adults with active disease defined by a pediatric Crohn's disease activity index of >7.5 or Harvey-Bradshaw index of ≥4	Prospective, open-label trial	*n* = 47 (7 used CDED without PEN)	6 wk	Response and remission were obtained in 37 (78.7%) and 33 (70.2%) patients, respectively. Remission was obtained in 70% of children and 69% of adults. Normalization of previously elevated CRP occurred in 21 of 30 (70%) patients in remission.
CDED+PEN vs EEN (34)	Children with CD with short duration of mild to moderate activity, mostly naive to treatment and with small bowel involvement (noninflammatory stricture or resection)	Randomized, nonblinded	*n* = 74. Group 1: 40 received CDED + 50% PEN for 6 wk (stage 1), followed by CDED + 25% PEN from wk 7 to 12 (stage 2). Group 2: 34 received EEN in stage 1, followed by a free diet with 25% PEN in stage 2.	12 wk	At wk 12, in group 1, 75.5% achieved remission, and of those, 75.9% had a normal CRP, 87.5% sustained remission, and microbiome exhibited dominance of *Clostridia* and decrease of *Proteobacteria*. At wk 12, in group 2, 45.1% achieved remission, and of those, 47.6% had a normal CRP, 56% sustained remission, and microbiome reverted to the pretreatment composition. In both groups, fecal calprotectin levels dropped significantly.
SCD vs Mediterranean diet (43)	Adult patients with CD, mild to moderate	Randomized trial	*n* = 191, 99 on the SCD and 92 on the MD	12 wk	At 6 wk, 47% on the SCD and 44% on the MD achieved symptomatic remission with up to 35% showing reduction of fecal calprotectin levels. At wk 12, 42% and 40% on the SCD and MD, respectively, achieved or maintained symptomatic remission. Fecal calprotectin response was observed only in 26% and 8% of patients on the SCD and MD, respectively. No significant change in alpha diversity was observed. In both groups, reduction of Faecalibacterium prausnitzii, Eubacterium eligens, and Eubacterium rectale was detected along with increased abundance of Bacteroides vulgatus and *Enterobacteriaceae.*
Low FODMAP diet (100)	Adults with IBD in remission or with mild disease	Randomized trial		6 wk	Reduced disease activity and fecal calprotectin along with improvement of self-reported quality of life in patients on low FODMAP diet compared to patients on standard diet.
Low FODMAP vs sham control diet (98)	Adults with quiescent IBD	Randomized single-blind trial, placebo control trial	*n* = 52 IBD patients, 27 on low FODMAP diet, 25 on placebo diet	4 wk	Higher health-related quality of life scores and reduction of symptoms in half of patients on low FODMAP diet compared to control diet. Low abundance of Bifidobacterium adolescentis, Bifidobacterium longum, and Faecalibacterium prausnitzii, along with reduction of total concn of SCFAs in low FODMAP diet-treated patients.
Low-fat, high-fiber diet (LFD) vs improved standard American diet (iSAD) (97)	Patients with UC in remission or with mild disease	Parallel-group, crossover study. Patients were randomized to an LFD (10% of calories from fat) or an iSAD (35%–40% of calories from fat) for the first 4-wk period, followed by a 2-wk washout period, and then switched to the other diet for 4 wk.	*n* = 17	4 wk on each diet	All patients remained in remission throughout the study. Both diets increased self-reported quality of life. Patients showed decreased abundance of *Actinobacteria*, whereas the relative abundance of *Bacteroidetes* increased. LFD favored the abundance of Faecalibacterium prausnitzii.
IBD-AID (37)	Patients with CD or UC, mild to severe activity	Retrospective	*n* = 11	4 wk or more	All patients discontinued at least one of their prior IBD medications, and all patients had symptom reduction. Disease activity scores decreased significantly.
IBD-AID (120)	Patients with IBD in remission or with mild to severe disease	Prospective, open-label trial. First, 6-wk baseline period, followed by an 8-wk intervention period.	*n* = 19	8 wk	Consumption of prebiotics, probiotics, and beneficial foods correlated with increased abundance of *Clostridia (*namely, Roseburia hominis, Faecalibacterium prausnitzii, Eubacterium eligens, Fusicatenibacter saccharivorans) and *Bacteroides* (namely, Bacteroides dorei, Bacteroides ovatus, and Bacteroides vulgatus) species. Prebiotics and adverse foods have an inverse impact on cytokine levels (i.e., IL-6, IL-8, TNF-α): negatively and positively correlating with proinflammatory cytokines, respectively.

a CRP, C-reactive protein: ESR, erythrocyte sedimentation rate.

### Adults.

“What to eat?*”* is the most frequent question asked by IBD patients of their treating physicians. Only recently has diet been recognized as a cost-effective strategy to induce remission in adult patients with IBD (37, 38, 43, 97). See Table 1 for a summary of the trials discussed below.

A recent randomized trial that included interventions with either the SCD or the Mediterranean diet (MD) has demonstrated a remarkable effect of diet in inducing remission in adults with Crohn’s disease (43). After only 6 weeks, 47% of the patients on the SCD (*n* = 99) and 44% on the MD (*n* = 92) achieved symptomatic remission (Crohn’s disease activity index [CDAI], <150), with up to 35% showing reduction in fecal calprotectin (FC) levels (reduction of FC to <250 μg/g and by >50% from screening among those with a screening FC level of >250 μg/g). At week 12, 42% and 40% on the SCD and MD, respectively, achieved or maintained symptomatic remission. The fecal calprotectin response was observed only in 26% and 8% of patients on the SCD and MD, respectively (43). The authors reported no significant changes in microbiome diversity at 0, 6, or 12 weeks post-dietary treatment, with patients in either diet group having comparable richness and Shannon’s diversity indices (43).

The low fermentable oligosaccharides, disaccharides, monosaccharides, and polyols (low FODMAP) diet has also been tested to manage irritable bowel syndrome-like symptoms in patients with IBD (38, 98–101). A randomized 6-week trial comparing the low FODMAP diet with a standard diet showed that patients (either CD or UC) on the low FODMAP diet modestly reduced their disease activity while those on the standard diet experienced no change in symptoms. Moreover, fecal calprotectin decreased only in the patients adopting the low FODMAP diet (100).

For ulcerative colitis, a catered nutritious low-fat, high-fiber diet (LFD) has been shown to improve the overall quality of life, lower inflammatory markers, and decrease dysbiosis (97). Fritsch et al. carried out a parallel-group crossover study to compare the effect of an LFD (10% of calories from fat) with that of an improved standard American diet (iSAD), which included higher quantities of fruits, vegetables, and fiber than a typical SAD with 35 to 40% of calories from fat. Patients with UC in remission or with mild disease (*n* = 17) were randomized to either diet for 4 weeks; after a 2-week washout period, patients were switched to the opposite diet. Although there were no significant differences in disease activity scores after each dietary intervention, which were low at baseline (mean partial Mayo score of 0.9), all patients remained in remission during the trial. Based on the validated short IBD questionnaire and the 36-Item Short Form Health Survey scores, participants in either diet group experienced increased quality of life compared to baseline measurements. Inflammatory markers such as serum amyloid A decreased significantly only in participants on the LFD, while microbe-derived metabolite SCFAs, particularly acetate, significantly increased with either dietary treatment. The LFD prompted a significant increase in *Bacteroidetes* and a decrease in *Actinobacteria*. In comparison to participants on the iSAD, participants on the LFD showed a significant increase in *Faecalibacterium prausnitzi*, a potent butyrate-producing bacterium commonly depleted in patients with IBD (90, 102–113).

Olendzki et al. have designed the IBD anti-inflammatory diet, or IBD-AID, to revert dysbiosis in IBD patients (37, 45). The IBD-AID promotes the intake of probiotic foods (independent of commercial supplements) to promote the establishment of commensal bacteria, prebiotic foods to feed commensal bacteria, and beneficial foods to meet dietary requirements, and the replacement of adverse foods thought to foster pathogenic microbiota and induce gastrointestinal symptoms (26, 37, 45, 114–119). The IBD-AID can be prepared at home, might be healthfully consumed by the entire family long-term, and can be adapted to meet other nutritional concerns as needed. In a retrospective study, both Crohn’s disease (*n* = 8) and ulcerative colitis (*n* = 3) patients adopting the IBD-AID experienced a reduction of disease activity and lowered their medication intake after only 4 weeks on the diet. Of the Crohn’s disease patients, the disease activity at baseline measured with the Harvey-Bradshaw index (HBI) averaged 11 (range, 1 to 20), and after dietary intervention, the HBI averaged 1.5 (range, 0 to 3). The ulcerative colitis patients had a mean baseline disease activity score, measured by the modified Truelove and Witts severity index (MTLWSI), of 7 (range 6 to 8), and their mean follow-up score was 0. The mean decrease in the HBI was 9.5, and the mean decrease in the MTLWSI was 7 (37). After the IBD-AID intervention, patients exhibited an increased abundance of potent butyrate-producing Faecalibacterium prausnitzii, Eubacterium eligens, and Roseburia hominis (120), all of which are commonly depleted in patients with IBD (90, 102–113).

## MOLECULAR MECHANISM OF DIET-MICROBIOME INTERACTION LEADING TO AMELIORATION OF IBD

The precise knowledge of the mechanisms by which food affects IBD will catalyze personalized nutritional therapy, sensitivity to differences in IBD clinical manifestations, host genotype, gut microbiome composition, and genetic capabilities. Compiling *in vitro* and *in vivo* evidence has shed light on the mechanisms by which diet decreases manifestations of the disease. A recent review summarizes studies with models of diseases detailing mechanistic findings (121). Here, I focus on the diet-microbiome interactions that affect the epithelial barrier permeability and the immune response in IBD.

### Dysbiosis and the epithelial barrier.

Dysbiosis in patients with IBD features a depletion of the commensal *Clostridia* and an expansion of *Enterobacteriaceae* species known to impact barrier function (90, 103–113, 122–125). Disruption of the epithelial barrier in IBD leads to the breach of bacteria and foreign antigens from the lumen into the underlying mucosa (8, 126–128). Once the epithelial barrier is infringed, a potent inflammatory response is activated, furthering the epithelial damage (129). Barrier dysfunction precedes IBD onset (130–139). Moreover, intestinal permeability is a robust predictor of poor outcomes and disease recurrence (132, 140–143). The gut microbiome is crucial in supporting a functioning epithelial barrier. For instance, the microbiome-derived SCFAs acetate, propionate, and butyrate represent the primary energy source for enterocytes (144), can act on genes involved in tight junction to seal the paracellular space (14, 145–147), increase oxygen consumption in the intestinal epithelium, which in turn stabilizes the hypoxia-inducible factor (HIF), a transcription factor responsible for maintaining barrier integrity (148), and sets an anti-inflammatory tone in the gut mucosa (14–17).

Plant-based foods, rich in fiber, are linked to increased abundance of SCFA-producing bacteria in IBD patients (32, 149). Conversely, fiber-deprived diets can cause a dysfunctional epithelial barrier. Schroeder et al. showed that mice fed a Western-like diet (high-fat/low-fiber) have an increased mucosal permeability and a reduced growth rate of the mucus layer (150). Transplantation of the microbiome of mice fed with chow into the mice fed a Western-like diet restores mucosal permeability and mucus growth. This highlights the importance of diet-dependent changes of the microbiome in mucosal barrier integrity and permeability (150). In a separate study, a low-fiber diet caused mucosal epithelial erosion, which in turn promoted lethal fulminant colitis in mice (151). Mice on low-fiber diets showed an expansion of mucus-foraging bacteria, such as Akkermansia muciniphila (152) and Bacteroides caccae (153), in comparison to mice on fiber-rich diets. The expansion of these mucolytic bacteria resulted in a permeable, eroded, thinner epithelial layer, with mice exhibiting intestinal inflammation (i.e., neutrophil infiltration, shorter colon length) and increased susceptibility to *Citrobacter*-induced colitis (151).

A high-fat diet also impairs barrier function, leading to susceptibility to colitis. A study from Xie et al. demonstrated that the 3-week offspring of mice fed a high-fat diet during pregnancy and lactation exhibited an abnormal epithelial layer with shorter villi, decreased crypt depth, and reduced number of proliferating cells, which in turn led to a lower number of differentiated intestinal cells, in comparison to animals fed a low-fat control diet (154). These morphological defects were accompanied by increased barrier permeability (measured by fluorescein isothiocyanate [FITC] in the serum) and decreased expression of tight junction proteins claudin 1 (CLDN), CLDN3, ZO-1, and occludin. Similarly, patients with severe IBD exhibit a compromised epithelial barrier with low levels of tight junction proteins, specifically occludin (155–158).

Comparisons of the gut microbiome of the offspring also revealed striking differences. Offspring of mice fed a high-fat diet had a lower alpha diversity than controls, an increased abundance of Akkermansia muciniphila, *Peptostreptococcaceae*, and Streptococcus, and a decreased abundance of butyrate-producing bacteria such as *Lachnospiraceae incertae sedis* and *Prevotellaceae.* Not surprisingly, the offspring of mice fed a high-fat diet and treated with dextran sodium sulfate (DSS; a sulfated polysaccharide widely used to reproducibly induce experimental acute and chronic colitis) exhibited more severe colitis than their counterparts (154). Similarly, adult mice fed a high-fat diet exhibited a significantly reduced expression of the tight junction protein occludin, which in turn compromised the epithelial barrier, leading to translocation of endotoxin (159).

In an elegant animal study, researchers tested the effect of over 40 diets, with various concentrations, combinations, and sources of macronutrients, on the microbiome, intestinal permeability, and colitis severity (160). They found that mice on diets high in protein (sources included chicken, beef, and egg whites) had increased intestinal permeability, weight loss, and severe colitis than mice on high-fiber diets. The effect of a high-protein diet (particularly casein) on colitis severity was mediated by the gut microbiome, since a high-casein diet reduces survival in comparison to a low-casein diet in specific-pathogen-free (SPF) mice but not in germfree animals. Moreover, casein-driven changes in gut microbial density were significantly associated with the severity of colitis seen in mice. Conversely, fiber-rich diets (particularly, the soluble fiber psyllium) increased the survival of mice treated with DSS (by at least 15 days compared to those on high-protein diets) and reduced colitis severity and the disruption of the epithelial permeability. The effects of dietary psyllium on colitis severity were both dependent and independent of the microbiome (160).

Another important food component to consider is dietary emulsifiers (i.e., carboxymethylcellulose, polysorbate 80, carrageenan, etc.). Emulsifiers are a ubiquitous component of processed food with detrimental effects on barrier function (161–164). Mice with chronic exposure to dietary emulsifiers exhibit erosion of the intestinal mucus layer and, in consequence, an enrichment of mucosa-associated inflammation-promoting *Proteobacteria* species (162, 163). More recently, a study showed that emulsifiers not only alter the mucosa-associated microbiota but also directly induce the expression of bacterial virulence genes to trigger chronic inflammation in mice (165).

In sum, evidence in animal models supports the importance of fiber-rich/low-fat/low-protein, emulsifier-restrictive diets to strengthen epithelial barrier function, which can contribute to resistance to colitis.

### Dysbiosis and the immune response.

The immune signature of IBD features an exacerbated epithelial infiltration of innate immune cells (i.e., neutrophils, macrophages, and dendritic cells) accompanied by an excessive activation of effector T cells (Th1, Th2, Th17, and T follicular helper [Tfh] cells) and/or altered tolerance mechanisms mediated by regulatory T cells (Tregs). Particularly, forkhead box protein P3 (FOXP3^+^) regulatory T cells or FOXP3^+^ Treg cells, located in the gut lamina propria, function as key regulators of intestinal inflammation in IBD (166–170). It has been established that *Clostridia* species, missing in IBD patients, are responsible for the activation of potent anti-inflammatory FOXP3^+^ Treg cells (18, 171, 172). Although there is an overall increase of *Clostridia* in patients adopting some of the IBD-friendly diets aforementioned, the impact of the diet-dependent *Clostridia* enrichment on Treg activation has not been studied in IBD patients undergoing dietary treatment. Yet, this provides a plausible hypothesis as one of the mechanisms behind those diets.

In a recent study, Song et al. aimed to investigate the diet-microbiome interactions that lead to the induction of Tregs (173). They compared induction of Tregs in three groups of mice: (i) specific-pathogen-free (SPF) mice fed a nutrient-rich diet, (ii) SPF mice fed a minimal diet, and (iii) germfree mice fed a nutrient-rich diet (173). Lower induction of Tregs was observed in SPF mice fed a minimal diet and germfree mice on a nutrient-rich diet, supporting the notion that both a rich diet and a functional microbiome are necessary for Treg induction. Moreover, investigators demonstrated that specific combinations of murine primary and secondary BAs added to the drinking water of SPF mice fed a minimal diet and germfree mice on a nutrient-rich diet restored Treg induction comparable to that of SPF mice fed a nutrient-rich diet. Deletion of either gene involved in BA deconjugation (bile salt hydrolase [BSH]) or the entire BA metabolic pathway in *Bacteroides* reduced the Treg induction. When susceptibility to colitis was tested, SPF mice on the minimal diet—with a lower proportion of colonic Tregs—exhibited higher weight loss and more severe colitis than SPF mice fed a rich diet. Although supplementation of primary and secondary BAs increased Treg cell counts in SPF mice on the minimal diet, after colitis onset, BA supplementation did not improve colitis in these mice. This highlights the importance of an initial BA-activated Treg pool to confer resistance to chemically induced colitis (173).

Devkota et al. found that mice on a diet high in saturated milk fat promoted taurine conjugation of BAs in the liver, which in turn increased the availability of organic sulfur that can be used by the taurine-respiring sulfidogenic organism Bilophila wadsworthia (174). *B. wadsworthia* is considered a pathobiont with a high capacity to reduce sulfites. The expansion of this bacterium was associated with a proinflammatory TH1 immune response and with a greater number of genetically susceptible IL-10^−/−^ mice developing colitis. In support of these findings, data from human subjects have shown that consumption of highly saturated fats (mainly animal fat) significantly increases taurine conjugation of BAs, production of fecal sulfide, and abundance of *B. wadsworthia* (23, 175–177).

Similarly, Sinha et al. demonstrated that microbiome-dependent BA modification causes intestinal inflammation in murine models of colitis (178). Here, investigators performed metabolomic analyses of stools from ulcerative colitis-pouch patients and found that there was a reduction in secondary BAs in ulcerative colitis-pouch patients, namely, lithocholic acid (LCA) and deoxycholic acid (DCA), while the accumulation of primary BAs was significantly higher than that in a control group. This metabolic profile was accompanied by a reduction of *Ruminococcaceae* sp. (involved in secondary BA production [179, 180]). Mice supplemented with secondary BAs showed a significant decrease in chemokines and cytokines, linked to inflammation, and an overall reduction of intestinal inflammation. The authors tested a chemically induced model of colitis (DSS and 2,4,6-trinitrobenzenesulfonic acid [TNBS]) as well as T cell transfer (CD45RB^hi^) murine models of colitis supplemented with the secondary BAs LCA and DCA. In every model of colitis, they observed a reduction of colitis as measured by weight loss, colon length, gross colon morphology, leukocyte infiltration, histology, and fecal lipocalin 2 when treated with secondary BAs. This effect was abrogated in mice lacking the G protein-coupled receptor TGR5, a transmembrane BA receptor. It has been demonstrated that secondary BA TGR5 agonists, DCA and LCA, can inhibit the production of tumor necrosis factor alpha (TNF-α) in CD14^+^ macrophages (181). Of interest, TNF-α is a proinflammatory mediator that plays an integral role in the pathogenesis of IBD.

Microbiome-derived SCFAs, favored by fiber-rich diets, also play a fundamental role in mediating the immune tone. In mice, fiber intake during pregnancy increases levels of butyrate in the blood of the offspring with concomitant increased numbers of peripheral and thymic Treg (182). Moreover, it has been shown that EEN supplemented with a multifiber mix prompted an expansion of mucosal CD4^+^ Foxp3^+^ Tregs along with an increase in concentrations of total SCFAs, i.e., acetate, propionate, and butyrate, in colitis-susceptible mice (IL-10^−/−^) (183). In addition, fiber supplementation reduced disease pathology and restored barrier function in IL-10^−/−^ mice (183). In contrast, mice on a high-fat diet exhibited reduced thymocyte counts and increased apoptosis of developing T cell populations (184). Butyrate can also activate Treg function. By acting on Treg, microbiome-derived butyrate reduces levels of proinflammatory cytokines, including TNF-α, interleukin 6 (IL-6), IL-1β, and MCP1/CCL2 (185–187). Moreover, microbe-derived butyrate promotes monocyte-to-macrophage differentiation via histone deacetylase inhibition. The macrophages differentiated by the addition of butyrate showed an enhanced antimicrobial activity, which *in vivo* increased colonization resistance to enteropathogens (188).

*In vitro* studies have demonstrated that other SCFAs, specifically acetate and propionate, promote differentiation of naive CD4^+^ T cells into Th17 cells with concomitant induction of *IL-17A*, *IL-17F*, *RORα*, *RORγt*, *T-bet*, and *IFN-γ* (189, 190). These SCFA effects on T cells combine histone deacetylase inhibitor-dependent and -independent mechanisms (189, 190).

In sum, increased evidence points at the pivotal role of diet-dependent changes of the microbiome in the immune response leading to/preventing colitis.

## FUTURE CONSIDERATIONS: CULTURALLY TAILORED DIETARY INTERVENTION FOR UNDERREPRESENTED IBD PATIENTS

Historically, IBD is known to affect more people of Caucasian origin than other ethnic groups; however, there is emerging evidence that the prevalence of IBD in Hispanics may be increasing, along with that in the general U.S. population (191, 192). Currently, Hispanics and Latinx account for over 18% of the U.S. population (193, 194). Foreign-born Hispanics in the United States are diagnosed at an older age and present more cases of ulcerative colitis than U.S.-born Hispanics and non-Hispanic whites (195). A meta-analysis also showed that Crohn’s disease behavior between non-Hispanic whites and Hispanics is similar, but Hispanics had a tendency of less upper gastrointestinal involvement (196). Growing evidence demonstrates that Hispanics change their diet upon immigration to the United States, reporting low consumption of total vegetables, legumes, whole grains, and sea plant protein (197). Similarly, Asians, the fastest-growing racial group in the United States (194), have been increasingly diagnosed with IBD in the United States and around the world (198, 199). Recent studies have demonstrated that Asians exhibit different IBD clinical phenotypes, including ocular manifestations and more fistulizing perianal Crohn’s disease, than their Caucasian counterparts (198, 199). A comprehensive study confirmed that U.S. immigrants (of Asian origins) suffer an immediate loss of gut microbiome diversity along with a reduction of microbial capacity for fiber degradation (200). Instead, the microbiome of Asian immigrants is characterized by an enrichment of United States-associated bacterial strains displacing native strains along with the genetic capabilities (200). Like Hispanics, Asian immigrants rapidly change their diet upon arrival in the United States, which partially explains the shift in the microbiome seen in this population (200).

The lack of inclusion of underrepresented ethnic minorities in IBD studies has ignited efforts led by patient advocates and IBD specialists of South Asian descent, such as the South Asian IBD Alliance (SAIAI) (201). The alliance aims to promote “the need for culturally competent, evidence-based, patient-centric care via advocacy, education, and training” to improve the care of South Asian patients with IBD across the globe (201).

Despite the increased incidence of IBD in these minority groups, research aimed at these populations, including research on diet as therapy for IBD, is lacking. In addition, ethnic minority groups frequently experience low-quality care at hospitals due to a combination of factors, including lack of insurance, economic and language barriers, and racial bias in pain assessment and treatment recommendations, to name a few (202–204). Culturally tailored interventions can close the gap in the paucity of research and help improve health care equity and quality for minority populations with IBD (205–208).

Culturally tailored interventions are frequently implemented in the context of behavioral health trials, with proven success to encourage healthy behaviors (including healthy eating) and to address health disparities affecting minority groups with chronic diseases (206, 209–211). A recent meta-analysis of 33 culturally tailored trials highlighted three key aspects of successful interventions (212), as follows. (i) “Linguistic tailoring” aims to address not only the language but also the literacy needs of the target population. Moreover, linguistic tailoring should also consider the inclusion of bilingual staff to remove language barriers between patients/participants, research staff, and health care providers. (ii) “Sociocultural tailoring” aims to incorporate cultural values, unique experiences, religious beliefs, and behaviors of the target group. (iii) “Constituent-involving strategies” aims to build on a sense of collectivism and existing kinship networks by including members of the target community in the research and intervention activities, from actively participating in the study design to their involvement in delivering the intervention (212, 213).

In the case of nutrition, culturally tailored interventions also need to be adapted to the unique culinary preferences and access to foods of the target community. By doing so, the interventions will be relevant to understudied minority groups with high IBD prevalence and in need of attainable strategies to improve their quality of life.

Another challenge for culturally tailored dietary interventions is long-term compliance. Examples from data about dietary recommendations in Australia (214) demonstrate that it is not enough to solely suggest that people consume more beneficial foods. Therefore, culturally tailored dietary interventions also need to provide patients with culinary training aimed at building skills and confidence in food preparation in the kitchen (215).
